# Draft Genome Sequence of Priestia megaterium MWU16-30321, Isolated from a Cranberry Stem Gall in Massachusetts

**DOI:** 10.1128/mra.01052-22

**Published:** 2022-12-06

**Authors:** Keenan Stephens, Alisha Harrison, Scott Soby

**Affiliations:** a Biomedical Sciences, College of Graduate Studies, Midwestern University, Glendale, Arizona, USA; b College of Veterinary Medicine, Midwestern University, Glendale, Arizona, USA; SIPBS, University of Strathclyde

## Abstract

Priestia megaterium MWU16-30321 was isolated from a mixed bacterial culture in a cranberry stem gall in Massachusetts following a severe winter. The genome is 5,623,390 bp in size and putatively encodes indole-3-acetic acid acetyltransferase, a key enzyme in tryptophan-dependent indole-3-acetic acid production.

## ANNOUNCEMENT

Tumorous formations in cranberry (Vaccinium macrocarpon Ait.) can result from mixed bacterial infections of stem tissue following mechanical or frost injury to the epidermis (1–3). These hypertrophic and hyperplastic galls form in response to bacterially produced indole-3-acetic acid (IAA); this results in stem girdling that causes the death of reproductive meristems, thus reducing fruit yield. Priestia megaterium MWU16-30321 (basonym Bacillus megaterium de Bary [4]) was isolated from a single stem gall in a commercial cranberry bog in Carver, Massachusetts, in 2015 by spreading surface-sterilized gall tissue on nonselective medium. The isolate was transferred to King’s medium B (KMB) agar supplemented with 50 μg mL^−1^ each of cycloheximide and ampicillin, incubated at 26°C for 24 h, colony purified three times on KMB agar, and stored at −80°C in 34% glycerol. A population of MWU16-30321 was inoculated into overnight KMB broth cultures for genomic DNA isolation with a DNeasy blood and tissue kit (Qiagen, USA). An Illumina-compatible genomic DNA library was generated with a KAPA HyperPlus library preparation kit (Kapa Biosystems KK8514; Roche, USA). DNA was enzymatically sheared to ~500-bp fragments, end repaired, and A-tailed, Illumina-compatible adapters with unique indexes (00989130v2; Integrated DNA Technologies, Coralville, IA) were individually ligated to each sample, and the DNA was cleaned using KAPA Pure beads (Kapa Biosystems KK8002) and amplified with HiFi enzyme (Kapa Biosystems KK2502). The library was analyzed for fragment size with an Agilent TapeStation and quantified by quantitative PCR (qPCR) (KAPA library quantification kit KK4835 with QuantStudio v5 [Thermo Fisher Scientific]) before multiplex pooling and sequencing on an Illumina MiSeq system with a 2 × 250-bp flow cell. All kits used in this work were used according to the manufacturer’s instructions. Raw reads were assembled with Unicycler v0.4.8 (5) and polished with Pilon v1.23 (6) within the PATRIC Comprehensive Genome Analysis pipeline v3.6.12, using default settings except for the trim setting, which was set to true (7). Adapter trimming and quality control were provided as part of the PATRIC pipeline by Trim Galore v0.4.0 (https://www.bioinformatics.babraham.ac.uk/projects/trim_galore) and QUAST v5.0.2 (8). Genome sequences were annotated using RASTtk v1.073 (9) as part of the PATRIC pipeline.

The 5,623,390-bp genome was assembled into 104 contigs, with a G+C content of 37.65 mol%, an *N*_50_ value of 610,290 bp, and 130× coverage. The average read length was 235.51 bp, from 3,115,330 total reads. There were 4 predicted rRNA genes and 84 tRNA genes. Of the 6,086 predicted genes, 4,017 had functional assignments, including IAA acetyltransferase, a key enzyme in tryptophan-dependent IAA production that has been observed in several *Bacillus* species (10–13).

MWU16-30321 was placed with high confidence in the species P. megaterium by phylogenetic analysis using the Type (Strain) Genome Server (TYGS) v342 (14). The digital DNA-DNA hybridization (dDDH) value (formula d_4_) was 94.2% with respect to P. megaterium ATCC 14581^T^ (GenBank accession number FOPA01000000).

### Data availability.

The whole-genome shotgun project has been deposited in DDBJ/EMBL/GenBank under BioProject accession number PRJNA765055, with BioSample accession number SAMN30839142, genome accession number JAOTPB000000000, and SRA accession number SRR18445423. The version described in this paper is JAOTPB010000000. RASTtk annotation is available under open license at Zenodo (https://doi.org/10.5281/zenodo.6415975).

## References

[1] Graham B, Castañeda T, Harrison A, Soby S. 2022. Draft genomic sequences of four *Pseudomonas* spp. and a *Xanthomonas* sp. from cranberry stem galls. Microbiol Resour Announc 11:e00999-21. doi:10.1128/mra.00999-21. 35230127 PMC8928770

[2] Vasanthakumar A, McManus PS. 2004. Indole-3-acetic acid-producing bacteria are associated with cranberry stem gall. Phytopathology 94:1164–1171. doi:10.1094/PHYTO.2004.94.11.1164. 18944451

[3] Best VM, Vasanthakumar A, McManus PS. 2004. Anatomy of cranberry stem gall and localization of bacteria in galls. Phytopathology 94:1172–1177. doi:10.1094/PHYTO.2004.94.11.1172. 18944452

[4] Gupta RS, Patel S, Saini N, Chen S. 2020. Robust demarcation of 17 distinct *Bacillus* species clades, proposed as novel *Bacillaceae* genera, by phylogenomics and comparative genomic analyses: description of *Robertmurraya kyonggiensis* sp. nov. and proposal for an emended genus *Bacillus* limiting it only to the members of the Subtilis and Cereus clades of species. Int J Syst Evol Microbiol 70:5753–5798. doi:10.1099/ijsem.0.004475. 33112222

[5] Wick RR, Judd LM, Gorrie CL, Holt KE. 2017. Unicycler: resolving bacterial genome assemblies from short and long sequencing reads. PLoS Comput Biol 13:e1005595. doi:10.1371/journal.pcbi.1005595. 28594827 PMC5481147

[6] Walker BJ, Abeel T, Shea T, Priest M, Abouelliel A, Sakthikumar S, Cuomo CA, Zeng Q, Wortman J, Young SK, Earl AM. 2014. Pilon: an integrated tool for comprehensive microbial variant detection and genome assembly improvement. PLoS One 9:e112963. doi:10.1371/journal.pone.0112963. 25409509 PMC4237348

[7] Wattam AR, Davis JJ, Assaf R, Boisvert S, Brettin T, Bun C, Conrad N, Dietrich EM, Disz T, Gabbard JL, Gerdes S, Henry CS, Kenyon RW, Machi D, Mao C, Nordberg EK, Olsen GJ, Murphy-Olson DE, Olson R, Overbeek R, Parrello B, Pusch GD, Shukla M, Vonstein V, Warren A, Xia F, Yoo H, Stevens RL. 2017. Improvements to PATRIC, the all-bacterial Bioinformatics Database and Analysis Resource Center. Nucleic Acids Res 45:D535–D542. doi:10.1093/nar/gkw1017. 27899627 PMC5210524

[8] Gurevich A, Saveliev V, Vyahhi N, Tesler G. 2013. QUAST: quality assessment tool for genome assemblies. Bioinformatics 29:1072–1075. doi:10.1093/bioinformatics/btt086. 23422339 PMC3624806

[9] Brettin T, Davis JJ, Disz T, Edwards RA, Gerdes S, Olsen GJ, Olson R, Overbeek R, Parrello B, Pusch GD, Shukla M, Thomason JA, Stevens R, Vonstein V, Wattam AR, Xia F. 2015. RASTtk: a modular and extensible implementation of the RAST algorithm for building custom annotation pipelines and annotating batches of genomes. Sci Rep 5:8365. doi:10.1038/srep08365. 25666585 PMC4322359

[10] Rashid U, Yasmin H, Hassan MN, Naz R, Nosheen A, Sajjad M, Ilyas N, Keyani R, Jabeen Z, Mumtaz S, Alyemeni MN, Ahmad P. 2022. Drought-tolerant *Bacillus megaterium* isolated from semi-arid conditions induces systemic tolerance of wheat under drought conditions. Plant Cell Rep 41:549–569. doi:10.1007/s00299-020-02640-x. 33410927

[11] Putrie RFW, Aryantha INP, Iriawati, Antonius S. 2021. The structure characteristic of IAA n-acetyl-transferase enzyme produced by two species of bacteria (*Bacillus subtilis* and *Bacillus amyloliquefaciens*). IOP Conf Ser Earth Environ Sci 762:e012054. doi:10.1088/1755-1315/762/1/012054.

[12] Wagi S, Ahmed A. 2019. *Bacillus* spp.: potent microfactories of bacterial IAA. PeerJ 7:e7258. doi:10.7717/peerj.7258. 31372316 PMC6659656

[13] Shao J, Li S, Zhang N, Cui X, Zhou X, Zhang G, Shen Q, Zhang R. 2015. Analysis and cloning of the synthetic pathway of the phytohormone indole-3-acetic acid in the plant-beneficial *Bacillus amyloliquefaciens* SQR9. Microb Cell Fact 14:130. doi:10.1186/s12934-015-0323-4. 26337367 PMC4558970

[14] Meier-Kolthoff JP, Göker M. 2019. TYGS is an automated high-throughput platform for state-of-the-art genome-based taxonomy. Nat Commun 10:2182. doi:10.1038/s41467-019-10210-3. 31097708 PMC6522516

